# Asthma prevalence, knowledge, and perceptions among secondary school pupils in rural and urban coastal districts in Tanzania

**DOI:** 10.1186/1471-2458-14-387

**Published:** 2014-04-23

**Authors:** Meshack Shimwela, Julius Chacha Mwita, Michael Mwandri, Godfrey Mutashambara Rwegerera, Yohana Mashalla, Ferdinand Mugusi

**Affiliations:** 1 Amana Municipal Hospital, P.O Box 25411, Dar es Salaam, Tanzania; 2 University of Botswana, Private Bag 0022, Gaborone, Botswana; 3 Muhimbili University of Health and Allied Sciences, P.O Box 65000, Dar es Salaam, Tanzania

**Keywords:** Asthma, Rural, Urban, Pupils, Tanzania

## Abstract

**Background:**

Asthma is a common chronic disease of childhood that is associated with significant morbidity and mortality. We aimed to estimate the prevalence of asthma among secondary school pupils in urban and rural areas of coast districts of Tanzania. The study also aimed to describe pupils’ perception towards asthma, and to assess their knowledge on symptoms, triggers, and treatment of asthma.

**Methods:**

A total of 610 pupils from Ilala district and 619 pupils from Bagamoyo district formed the urban and rural groups, respectively. Using a modified International Study of Asthma and Allergies in Childhood (ISAAC) questionnaire, a history of “diagnosed” asthma or the presence of a wheeze in the previous 12 months was obtained from all the studied pupils, along with documentation of their perceptions regarding asthma. Pupils without asthma or wheeze in the prior 12 months were subsequently selected and underwent a free running exercise testing. A ≥ 20% decrease in the post-exercise Peak Expiratory Flow Rate (PEFR) values was the criterion for diagnosing exercise-induced asthma.

**Results:**

The mean age of participants was 16.8 (±1.8) years. The prevalence of wheeze in the past 12 months was 12.1% in Bagamoyo district and 23.1% in Ilala district (p < 0.001). Self-reported asthma was found in 17.6% and 6.4% of pupils in Ilala and Bagamoyo districts, respectively (p < 0.001). The prevalence of exercise-induced asthma was 2.4% in Bagamoyo, and 26.3% in Ilala (P < 0.002). In both districts, most information on asthma came from parents, and there was variation in symptoms and triggers of asthma reported by the pupils. Non-asthmatic pupils feared sleeping, playing, and eating with their asthmatic peers.

**Conclusion:**

The prevalence rates of self-reported asthma, wheezing in the past 12 months, and exercise-induced asthma were significantly higher among urban than rural pupils. Although bronchial asthma is a common disease, pupils’ perceptions about asthma were associated with fear of contact with their asthmatic peers in both rural and urban schools.

## Background

Asthma is one of the most common childhood disorders that is associated with significant morbidity [1]. There is wide variation in asthma prevalence across countries throughout the world [2]. In Phase One of the ISAAC study, the prevalence of asthma symptoms ranged from less than 5% in developing countries to more than 20% in developed countries [2]. The prevalence of asthma has also been increasing over the past few decades [3-5], with the highest increase seen among children and young adults living in the inner cities and in regions where the prevalence had previously been low [2,5,6]. This increase has mainly been attributed to the increasing urbanization in developing countries, including those in Africa [7,8]. The traditional rural lifestyle and early childhood exposure to infectious agents in rural areas are believed to be protective against asthma and allergic diseases [9]. Infection in early childhood, facilitated by unhygienic conditions within families and communities, has been shown to prevent the development of allergic diseases, including asthma [10]. Urbanization on the other hand is associated with a reduction in the frequency of childhood infections, due to the increased use of antibiotics, improved personal and public hygiene, and changes in diet [11]. As a result of these differences in frequency of early childhood infections, there is a lower prevalence of asthma in rural areas as compared to urban areas. In addition, urbanisation is associated with increased environmental pollution, sedentary lifestyle, obesity and unhealthy diet; factors associated with increased risk of asthma [11-13]. There are also variations in beliefs and perceptions on asthma, due to differences in the level of education between urban and rural areas [14]. These differences not only affect treatment of asthma, but also contribute to the increase in morbidity and mortality of the disease [14]. This study sought to estimate the prevalence of asthma, and to assess perceptions with regards to asthma among secondary school pupils in the rural and urban coast of Tanzania.

## Methods

### Study design

This was a cross-sectional study that was conducted to determine the prevalence of asthma and information about symptoms, triggers, and perceptions of asthma among form I – IV secondary school pupils in the Ilala and Bagamoyo districts in Tanzania.

### Study population

The study involved secondary school pupils from Ilala and Bagamoyo districts, both of which are located along the Indian Ocean. Ilala is one of the three districts of Dar es Salaam, Tanzania’s largest commercial city (population 634,924) [15], and one of the most industrialized and polluted districts in the country. Bagamoyo is a rural colonial town with a population of 228,967 [15], which has no industries and depends largely on farming and fishing. According to the Ministry of Education and Culture, there were 18 and 9 secondary schools located in Ilala and Bagamoyo, respectively. With simple random selection, six secondary schools were selected from each district, and from these schools 27 pupils from each of the four classes were randomly selected. To minimise the influence of other disease conditions with symptoms similar to asthma, pupils with known chronic heart diseases were excluded from the study. The sample size was calculated using a sampling error of 2%, 90% power, and anticipated population prevalence of asthma of 6% [16]. These parameters gave a minimum sample size of 542 pupils from each district, which was increased by 20% to a total of 1301 pupils to account for non-response.

### Procedures and outcomes

The data were collected in 2007 using a modified ISAAC questionnaire [17]. Additional questions on pupils’ perceptions regarding asthma and their knowledge of symptoms and triggers of asthma were included in the questionnaire. The original English version of the questionnaire was translated into Swahili by two independent health professionals. In order to check for any differences when compared with the original English version, a back translation into English was then done by another qualified translator. The Swahili questionnaire was then piloted among 25 pupils in Ilala district who were selected from a school which was not part of the studied population. It was administered by researchers to assess if all of the respondents interpreted questions in the same way, whether the Swahili interpretation for words such as “wheeze” and “asthma” would be understood by the pupils in the study, and whether each question measured what the study was intended to measure. No focus group discussion was done, and the information from the pilot was used to develop the final Swahili version of the questionnaire.

Permissions to involve pupils in the study were initially sought from the district educational authorities, head-teachers, and class teachers. During the initial visit to each school, pupils were informed about the study objectives and procedures. Pupils aged 18 years or older signed consent forms themselves, if they agreed to participate in the study. For pupils less than 18 years old and not living in the school campuses, information sheets explaining the study’s purpose and procedures were provided to their parents/guardians. Parents/guardians were requested to give signed consent if they agreed to allow their children to participate in the study. For those pupils living in hostels, a witnessed consent from their teachers (matrons/patrons) was used. In addition, all pupils younger than 18 years were required to sign assent forms. All information sheets, consent and assent forms, and validated questionnaires were in Swahili. Subsequent visits were arranged to collect information on consented pupils. Of 1301 pupils selected for interview, 1229 (94.5%) agreed to participate, and a total of 619 pupils from Bagamoyo and 610 pupils from Ilala were enrolled. Seventy two pupils did not participate in the study for various reasons. Of these, 6 pupils had known cardiac diseases. None of them had a chronic respiratory disease or asthma. In addition, sixty six pupils were excluded from the study for various reasons including acute illnesses during the study days (malaria-2 pupils, gastroenteritis-2 pupils and pneumonia-3 pupils) and either refusal to participate or non-availability at school during the study days (59 pupils).

Using self-administered questionnaires, a history of “diagnosed” asthma or the presence of wheezing in the prior 12 months was obtained from all the pupils studied. Each pupil’s age was documented, and anthropometric data including height and weight, were measured. Although all pupils knew their dates of birth, this information was verified by checking school records. Their standing heights were measured using a specially designed portable stadiometer to the nearest 0.1 centimetre. Body weight was determined to the nearest 0.1 kg with a calibrated digital scale (SECA, Type 803, United Kingdom), with the subject standing and wearing light clothes. Peak expiratory flow rates (PEFR) were assessed using Micro-Spirometers (Micro Medical Limited England). After a group demonstration and participant practice using the peak flow meter, each subject was requested to perform three peak flows, from which the highest value was recorded as the pupil’s PEFR.

Pupils without self-reported asthma or wheeze were subsequently exercised by freely running for six minutes on a plane football ground. Within five to ten minutes after exertion, each pupil performed at least three PEFR measurements, supervised by the same observer. The measurement with the highest reading was recorded. The percentage fall in the post-exercise PEFR was then calculated as follows:

$$\left\{\left(\mathrm{Pre}‒\mathrm{exercise}\;\mathrm{PEFR}‒\mathrm{Post}\;\mathrm{exercise}\;\mathrm{PEFR}\right)/\mathrm{Pre}‒\mathrm{exercise}\;\mathrm{PEFR}\right\}\times 100$$

Exercise-induced asthma was defined as a fall of post-exercise PEFR by ≥ 20% [18].

The study was approved by the Ethics Committee of the Muhimbili University College of Health and Allied Sciences (MUCHS).

### Statistical analysis

Data were entered, cleaned, and analysed using the Statistical Package for Social Sciences version 10.0 (SPSS Inc., Chicago, IL). Means and standard deviation were calculated for quantitative data. We used the Chi-squared test to determine the associations between categories. Pearson correlation was used to find the significant relationship between PEFR and height of a pupil. A p-value < 0.05 was considered statistically significant.

## Results

### Characteristics of the study population

A total of 1229 pupils participated in the study. The mean (SD) age of participants was 16.8 (±1.8) years, 68% of them being less than 17 years old, with the Ilala district having a significantly younger group than Bagamoyo (Table 1). Overall, there were more boys (59.2%) than girls involved in the study.

**Table 1:** Age and sex distribution of pupils in the two districts

**Age (yrs)**	**Bagamoyo**		**Ilala**		
	**Male n (%)**	**Female n (%)**	**Male n (%)**	**Female n (%)**	**Total n (%)**
≤15	64(13.6)	25(16.9)	93(36.3)	131(37.0)	313(25.5)
15-17	169(35.9)	80(54.1)	100(39.1)	174(49.2)	523(42.6)
17-19	165(35.0)	38(25.7)	48(18.8)	46(13.0)	297(24.2)
19+	73(15.5)	5(3.4)	15(5.9)	3(0.9)	96(7.8)
Total	471(38.3)	148(12.1)	256(20.8)	354(28.8)	1229(100)

### Prevalence of self-reported asthma and wheeze

The prevalence of self-reported asthma and wheeze among pupils in the studied areas is presented in Table 2. The overall prevalence of self-reported asthma was 11.8%. Pupils from the schools in Ilala district had a higher prevalence of self-reported asthma as compared to those in Bagamoyo (17.1% versus 6.6%, p < 0.001). The majority of pupils in Bagamoyo (58.6%) and Ilala (61.1%) knew whether or not they had a prior diagnosis of asthma from their parents, and about a third of them (29.3% in Bagamoyo and 30.6% in Ilala) knew this information from healthcare workers.

**Table 2:** Prevalence of wheeze and self-reported asthma in Bagamoyo and Ilala districts

**District**	**Sex**		
**Bagamoyo (n = 619)**	**Male n (%)**	**Female n (%)**	**P-value**
Self-reported asthma	30 (6.4)	11 (7.4)	0.500
Wheeze in the prior 12 months	54 (11.5)	21 (14.2)	0.150
Wheeze in the prior12 months among patients with self-reported asthma	24 (5.1)	8 (5.4)	0.898
**Ilala (n = 610)**			
Self-reported asthma	45 (17.6)	59 (16.7)	0.704
Wheeze in the prior 12 months	58 (22.7)	83 (23.5)	0.734
Wheeze in the prior 12 months among patients with self-reported asthma	33 (12.9)	41 (11.6)	0.486

Overall, 17.6% (n = 216) of pupils reported to have had wheeze in the previous 12 months. It was reported in 75/619 (12.1%) pupils in Bagamoyo and 141/610 (23.1%) pupils in Ilala (p < 0.001). Of the pupils with self-reported asthma, 106/216 (49.1%) reported having had wheeze in the past 12 months. Wheezing in the past 12 months was 32/75 (42.7%) among asthmatic pupils from Bagamoyo and 74/141 (52.5%) pupils from Ilala (P = 0.17). There was no gender difference in the self-reported asthma and wheezing over the past 12 months.

In addition to wheezing, other symptoms reported by pupils in both districts included shortness of breath, cough, and chest tightness. In Bagamoyo, shortness of breath, cough and chest tightness were reported in 19.4%, 20.8% and 12% of the pupils, respectively. Similarly, pupils in Ilala reported shortness of breath (24%), cough (15.8%) and chest tightness (24.4%).

### Peak expiratory flow rate (PEFR)

The mean resting PEFR for male pupils was 478.05 ± 77.99 l/min in Ilala and 484.04 ± 79.18 l/min in Bagamoyo (p < 0.33). Mean resting rates for female pupils were 400.00 ± 57.00 l/min and 393.92 ± 62.02 l/min in Ilala and Bagamoyo, respectively (p < 0.28). In both study areas, there was a significant positive correlation between height and (P = 0.01). For every one meter increase of height there was an increase in PEFR of 0.537 l/s and 0.446 l/s for Ilala and Bagamoyo, respectively. The correlation was R = 0.7 and R = 0.5 for Ilala and Bagamoyo, respectively.

### Prevalence of exercise-induced asthma

Using reductions in PEFR of both 20% and 15%, exercise-induced asthma was more prevalent among urban than rural pupils. With 15% PEFR reduction, asthma was diagnosed in 4.3% and 7.2% of pupils Ilala and Bagamoyo, respectively (P = 0.044). Similarly, using a 20% drop in PEFR to define exercise-induced asthma, the prevalence of asthma was significantly higher (P = 0.002) in Ilala (6.3%) than Bagamoyo (2.4%).

### Knowledge of asthma triggers

Table 3 lists the common triggers for asthma mentioned by the pupils. Over half of healthy pupils, 69.8% and 53.8% (p < 0.001) from Ilala and Bagamoyo districts, respectively, were aware of asthma as a disease. Dust and cat hair/fur were the commonest asthma triggers reported. About one fifth (21.3%) of pupils in Ilala and 16.3% of pupils in Bagamoyo reported avoidance of the triggers as the means of preventing asthma attacks. Several modes of treatment were reported, including hospital medicines (58% and 70%); traditional herbs (33.3% and 17.6%), and honey (4% and 7%) for Bagamoyo and Ilala, respectively. Other treatments mentioned were raw eggs, induced vomiting, better ventilation, and heavy clothing.

**Table 3:** Knowledge of the triggers of asthma among pupils in the two districts

**Reported trigger factors**	**Bagamoyo n (%)**	**Ilala n (%)**	**P Value**
Dust/kapok tree dust	67 (10)	117 (17.5)	< 0.0001
Cat hairs/fur	68 (10.2)	80 (12.0)	0.297
Cold whether	29 (4.3)	37 (5.6)	0.316
Prawns	18 (2.7)	13 (2.0)	0.468
Exercise	9 (1.3)	17 (2.6)	0.118
Fumes/perfumes	3 (0.5)	11 (1.6)	0.034
Aspirin	4 (0.6)	5 (0.8)	0.753
Cigarette smoke	5 (0.75)	4 (0.6)	1.0
Cough	0	4 (0.6)	0.062
Don’t know	467 (69.7)	400 (60.0)	<0.001
Total	670 (100)	668 (100)	

### Perceptions about asthma

Table 4 shows the perceptions of asthma among the study’s participants. Non-asthmatic pupils feared sleeping, playing, studying, and eating with their asthmatic peers, a finding which suggests that the non-asthmatic pupils believed that asthma is infectious.

**Table 4:** Perceptions about asthma among pupils in selected secondary schools in Ilala and Bagamoyo districts

**Perceptions about asthma**	**Bagamoyo n (%)**	**Ilala n (%)**	**P- value**
Fear playing together	128 (20.7)	78 (12.8)	0.0001
Fear studying together	95 (15.3)	59 (9.7)	0.003
Fear eating together	117 (18.9)	82 (13.4)	0.009
Fear sleeping in the same room	169 (27.3)	114 (18.1)	0.0001

## Discussion

Asthma is globally one of the most common childhood chronic disorders associated with significant hospitalization and school absenteeism [1,19]. Therefore, it is important for countries to establish reliable data on the prevalence of childhood asthma, and to institute effective interventions to reduce the economic and social costs associated with childhood asthma.

This study compared the prevalence of childhood asthma among secondary school pupils in both rural (Bagamoyo) and urban (Ilala) districts along the Indian Ocean coastline of Tanzania. The study has shown that asthma was more prevalent among pupils living in urban areas than among those living in rural areas. Although the magnitude of differences varied, this rural–urban difference in prevalence has also been reported in other studies [8,9,20,21]. The 6.6% prevalence for self-reported asthma in the rural pupils in this study is comparable to that reported from other parts of Africa, India, and tropical countries [2,6]. Similarly, the high prevalence of 17.1% for self-reported asthma in the urban pupils from Ilala is comparable with that reported from both the western world and other African cities [2,8,22]. In the ISAAC study, similar prevalence rates were reported among 13 to 14 year-old pupils in Cape Town (20.3%), Polokwane (18.0%), Reunion Island (21.5%), Brazzaville (19.9%), Nairobi (18.0%), Urban Ivory Coast (19.3%) and Conakry (18.6%) [22]. However, the reported prevalence rates for both urban and rural self-reported asthma in our study are higher than the prevalence of 1.9–5.2% that was previously estimated in Tanzania in 2004 [16]. This discordancy is probably due to time and methodological differences between the two studies. Nonetheless, the prevalence of wheeze in the past 12 months for the rural pupils in the current study is comparable to that reported among 9–10 year old children in Northern Tanzania [23].

Exercise tests have been widely used in the diagnosis of asthma in children [21,24]. Although a negative test does not automatically rule out asthma, a drop in the peak flow rate of 20% of the resting rate is considered positive [18]. Falls in peak expiratory flow rates of 10%, 15% or even a 25% have also been used to define exercise-induced asthma in some studies [18,21,24]. The use of different cut-off values has not only contributed to the variation in the reported prevalence rates of exercise-induced asthma, but has also rendered comparison of findings from these studies problematic. Using a 20% fall in PEFR, the prevalence of asthma in rural Bagamoyo and urban Ilala is 2.3% and 6.4%, respectively. On the other hand, using a 15% fall in PEFR as the criteria for diagnosis of childhood asthma, the prevalence in our study increased to 4.3% and 7.2% for rural and urban areas, respectively. These rates are higher than the prevalence rates reported in a South African study that used a 15% drop in PEFR to define exercise-induced asthma and found prevalence rates of approximately 3% in urban children and 0.1% in rural children [21]. Similar to other studies, we found a rural–urban difference in the prevalence rates of exercise-induced asthma [7,21,24].

In this study, the majority of pupils had received information about their asthma status from parents and health facilities. A surprising finding of our study was that schools were not significant sources of the pupils’ information about causes and pathophysiology of asthma. The findings suggest that there are gaps in the secondary school curricula regarding asthma education, an issue that needs to be revisited.

Our study also reports varying perceptions about asthma among the studied pupils, suggesting pupil belief that asthma is an infectious disease. These perceptions may be related to our additional finding that non-asthmatic pupils fear playing, eating with, or sleeping in the same room with asthmatic pupils. These observed perceptions suggest inadequate or deficient knowledge about the causes and pathophysiology of asthma on the part of the study’s pupils. The non-asthmatic pupil’s fear of their peers with asthma was found to be more pronounced among rural rather than urban pupils, a finding that may be explained by differences in socio-cultural values and access to information about asthma. Parents have been reported to play an important role in shaping the health care behaviour and beliefs of pupils. However, most parents are likely to share their own cultural norms, prejudices, fears, and beliefs. Teachers on the other hand, because of their intense and prolonged contact with pupils during the school year, are in a better position to be an important source of information about asthma. Nonetheless, teachers may not be able to provide such valuable health information, because they teach according to standard curricula, which may not include information about asthma or because of their own ignorance and/or personal beliefs about the disease. In this regard, provision of correct information to parents and teachers about childhood asthma may alleviate the fears many pupils may have about this common childhood condition.

## Conclusion

The prevalence of self-reported asthma, wheeze, and exercise-induced asthma was lower in secondary school pupils in a rural area of costal Tanzania compared with similarly matched pupils in a costal urban area. In both studied populations, pupils had perceptions about asthma that suggested they believed it is an infectious disease, a belief that was associated with fear of contact with asthmatic pupils in rural, as opposed to urban pupils. As most of the pupils’ information on asthma came from their parents, the same perceptions may also be held by their communities at large. It is recommended that further studies on the knowledge and perceptions about asthma should be carried out on a larger scale, for the purpose of developing educational materials for schools and the general population. It is anticipated that provision of accurate information about asthma to pupils will empower communities to develop preventive strategies and effective management of asthma.

Although pupils in Ilala and Bagamoyo districts represent urban and rural settings, respectively, they may not represent pure urban and rural communities, because of frequent migration of pupils between rural and urban areas for schooling. In addition, these two districts are located along the coast of Tanzania, and the findings of this study may not be generalizable to mainland Tanzania. A further limitation of this study may be pupil unreliability regarding the recall of wheeze in the prior 12 months. Although a Swahili description of “wheeze” was provided before the administration of questionnaires, misinterpretation of this term is a possibility with some of the study’s pupils. The Swahili language lacks a word precisely equivalent to wheeze.

## Competing interests

The authors declare that they have no competing interests.

## Authors’ contributions

JM led the manuscript draft writing and incorporating the co-author’s comments. MS, JM, FM conceived the study and did analysis. MS, JM, FM, MM, GR and YM did literature search, manuscript drafting and review. All authors read and approved the final manuscript.

## Pre-publication history

The pre-publication history for this paper can be accessed here:

http://www.biomedcentral.com/1471-2458/14/387/prepub
